# Identification, Typing, and Drug Resistance Analysis of *Escherichia coli* in Two Different Types of Broiler Farms in Hebei Province

**DOI:** 10.3390/ani13203194

**Published:** 2023-10-13

**Authors:** Chuncai Liang, Huan Cui, Ligong Chen, Hailong Zhang, Cheng Zhang, Juxiang Liu

**Affiliations:** College of Veterinary Medicine, Hebei Agricultural University, Baoding 071000, China

**Keywords:** broiler, antibiotic resistance, multilocus sequence typing

## Abstract

**Simple Summary:**

Antibiotic resistance is a global health crisis faced by humanity. In response to this crisis, in 2018, China released the National Pilot Work Program for Action to Reduce the Use of Veterinary Antimicrobial Drugs. Pilot work to reduce the use of antimicrobial drugs was carried out on livestock and poultry scale farms across the country, and the farms implementing the reduction in antimicrobial drug use were accepted as meeting the standards by the “Evaluation Standards and Methods for Reducing the Use of Veterinary Antimicrobial Drugs on Livestock and Poultry Farms”. To evaluate the effect of antimicrobial drug reduction on antibiotic resistance, we selected six broiler farms in Hebei Province for the first time, four of which implemented antimicrobial drug reduction and passed national acceptance (SFs), and the other two did not implement antimicrobial drug reduction (NSFs). By collecting cloacal swabs from healthy broilers, isolating and characterizing *Escherichia coli (E. coli*), and determining their resistance phenotypes, resistance profiles, and genotypes to 16 antimicrobial drugs, as well as ST typing of the isolates, we compared the differences in resistance and the genetic evolutionary relationships of two different types of broiler farms in Hebei Province, China.

**Abstract:**

Hebei Province is an important area for breeding broiler chickens in China, but the antimicrobial resistance and prevalence of *Escherichia coli* (*E. coli*) are still unclear. A total of 180 cloacal samples from broiler farms in Hebei Province were collected and used for the isolation and identification of *E. coli*. The isolates were subjected to resistance phenotyping, resistance profiling, and genotyping, and some multiresistant strains were subjected to multilocus sequence typing (MLST). The results showed that 175 strains were isolated. Among both types of broiler farms, the ampicillin resistance rate was the highest, and the meropenem resistance rate was the lowest. Serious multiresistance was present in both types of broiler farms. Thirty strains of multidrug-resistant *E. coli* were typed by MLST to obtain a total of 18 ST types, with ST10 being the most prevalent. This study was to simply analyze the antimicrobial resistance and prevalence of *E. coli* in broiler chickens in Hebei Province after the implementation of the pilot work program of action to reduce the use of veterinary antimicrobials in standard farms (SFs) and nonstandard farms (NSFs). This study will provide a research basis and data support for the prevention and control of *E. coli* in Hebei.

## 1. Introduction

*Escherichia coli* (*E. coli*) is a gram-negative bacterium in the Enterobacteriaceae family of bacteria that is widely present throughout the entire feeding chain of chicken farms, in chicken houses, in the air, and even in the food chain, that can cause significant economic loss to the livestock industry and seriously affect global public health security [1,2,3,4]. Currently, there is still no effective vaccine for the prevention of *E. coli* infection, and the use of antibiotics remains the primary choice. Antibiotics are extensively used in livestock and poultry farming, usually for the treatment and prevention of bacterial diseases, and there is no complete substitute for them [5,6]. However, the misuse and overuse of antibiotics and insufficient research and development of new antibiotics have led to the emergence of antibiotic resistance, posing significant threats to livestock product safety and presenting a potential health risk to the public [7,8]. Moreover, through continuous monitoring and research by domestic and foreign researchers, it has been found that the resistance of *E. coli* is increasing and rapidly spreading, making the prevention and control of *E. coli* infections more challenging [9,10,11]. Antibiotic resistance is closely related to the misuse and duration of use. Over time, *E. coli* has demonstrated a significant level of resistance to commonly used antibiotics [12,13].

The rise of antimicrobial resistance (AMR) has made the misuse of antibiotics a major cause for concern [14,15]. The frequent administration of antibiotics to poultry is especially alarming, as it has led to a significant increase in resistance rates, posing a serious threat to public health. Moreover, the misuse of antibiotics is closely linked to AMR. In 2015, the World Health Organization (WHO) called for the rational and standard use of medically necessary antimicrobial drugs in both humans and animals and released the AMR Global Action Plan [16,17]. China currently holds the position of being the leading producer and consumer of antibiotics globally. Moreover, more than 50% of the total annual usage of antibiotics can be attributed to the livestock and poultry industry in China, which has resulted in significant challenges regarding antibiotic resistance [18]. To this end, China has taken a series of measures to control the use of antibiotics in animal husbandry. In 2016, China released the “National Action Plan to Curb Bacterial Resistance (2016–2020)”. In 2018, China implemented the National Pilot Work Program for Action to Reduce the Use of Veterinary Antimicrobial Drugs and carried out standard acceptance of farms implementing reduced use of antimicrobial drugs by the “Evaluation Criteria and Methods for Evaluating the Effectiveness of Reducing the Use of Veterinary Antimicrobial Drugs on Livestock and Poultry Farms”. These efforts are aimed at guiding farms in the rational use of antimicrobial drugs and monitoring bacterial resistance of animal origin to effectively curb the problem of bacterial resistance caused by the misuse of antibiotics and improve the effectiveness of the use of antimicrobial drugs. It is noteworthy that farms that meet or exceed the provincial evaluation standards can obtain the “Standardized Farm with Reduced Antibiotic Use for Animal Production” label. Despite this, there has been little research carried out on the impact of the antibiotic resistance of bacteria in SFs. Therefore, by monitoring antibiotic resistance genes, assessing the resistance status of antimicrobial drugs, and conducting multilocus sequence typing, we can gain a basic understanding of the impact of antibiotic resistance and epidemiological characteristics of *E. coli* strains after action to reduce the use of veterinary antimicrobial drugs. This will contribute to the promotion of appropriate utilization of antibacterial drugs and the effective control of *E. coli* infections.

In this experiment, cloacal swabs were collected from two different types of broiler farms in Hebei Province. *E. coli* was isolated and identified, and drug susceptibility testing and resistance gene detection were carried out to analyze the impact of the reduced use of veterinary antimicrobials on the prevalence of antibiotic resistance and, thus, to assess the impact of the reduced use of antimicrobials on antibiotic resistance in Hebei Province, China. At the same time, multilocus sequence typing was performed on some multidrug-resistant strains to analyze the genetic evolution and explore the genetic characteristics between two types of broiler farms in Hebei Province, China, which will further provide a basis for guiding the rational use of clinical medication and controlling the emergence and spread of antibiotic resistance, which is of great significance in public health.

## 2. Materials and Methods

### 2.1. Sample Source

**Figure 1:**
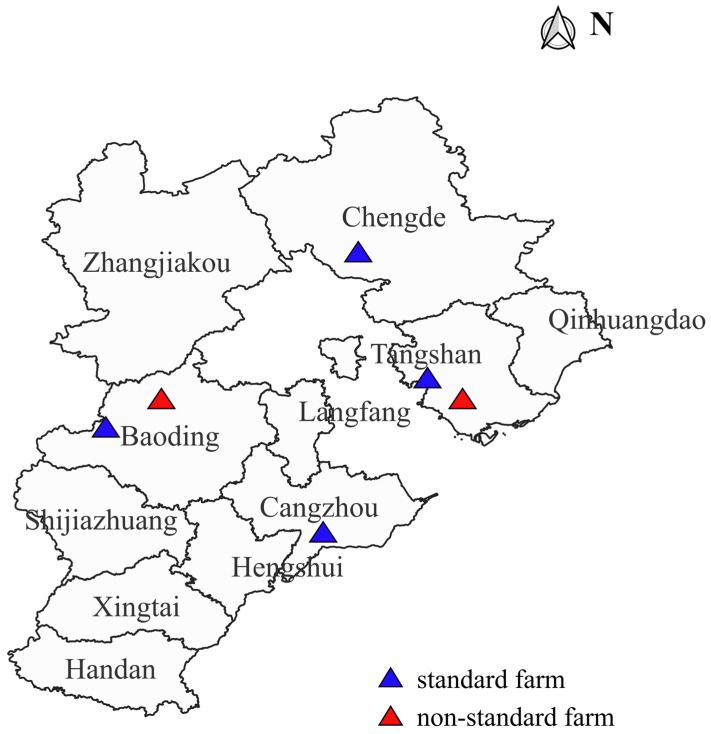
The distribution of broiler farms in Hebei Province, China.

A total of 180 cloacal swabs were collected from six broiler farms located in the Baoding, Chengde, Tangshan, and Cangzhou regions in Hebei Province, including four SFs and two NSFs (Figure 1). The six broiler farms’ details are shown in Table S1. All cloacal samples were collected from healthy broilers, and 30 chickens were randomly selected from each broiler farm. The samples were kept in an ice box and sent to the laboratory in a timely manner. Information on antibiotic treatment for the six sampled farms during the study period is shown in Table S2.

### 2.2. Main Reagents

Normal Nutrient Agar, MacConkey Agar, and Eosin Methylene Blue Agar (EMB) were purchased from Beijing Aoboxing Biotechnology Co., Ltd. (Beijing, China).; DL 2000 DNA Marker was purchased from TAKARA; 2×Taq PCR Mix was purchased from Beijing Yuanchen Technology Co., Ltd. (Beijing, China). Gentamicin (GEN, 10 μg), spectinomycin (SPT, 100 μg), tetracycline (TE, 30 μg), ampicillin (AMP, 10 μg), ceftazidime (TAZ, 30 μg), ofloxacin (FOX, 5 μg), cotrimoxazole (SXT, 1.25/23.75 μg), and other drug-sensitive Discs were purchased from Hangzhou Binhe Microbiological Reagent Co., Ltd. (Hangzhou, China).; amoxicillin/clavulanic acid (AMC, 20/10 μg), florfenicol (FFC, 30 μg), meropenem (MEM, 10 μg), enrofloxacin (ENR, 5 μg), and sulfisoxazole (SIZ, 250/300 μg) were purchased from Hangzhou Microbial Reagent Co., Ltd. (Hangzhou, China).; apramycin (APR, 30 μg), polymyxin (CT, 10 μg), ceftiofur (EFT, 30 μg), and mequindox (SPI, 30 μg) were purchased from Yinuokang Technology Development Co., Ltd. (Tianjin, China). *Escherichia coli* quality control strain (25922, ATCC, USA) was purchased from Beina Chuanglian Biotechnology Co., Ltd. (Beijing, China).

### 2.3. Isolation and Identification of E. coli

The collected cloacal swabs were streaked onto MacConkey (MAC) and eosin methylene blue agar (EMB) and cultured at 37 °C for 15 h. Following three rounds of purification, purified single colonies were selected for Gram staining, and the morphological characteristics of the isolated strains were observed under a microscope. The *E. coli* strains were confirmed by PCR detection of the *16S rRNA* gene using the following primers:

*16S rRNA*-F, 5′-ATCATGGCTCAGATTGAACG-3′,

*16S rRNA*-R, 5′-CCAGTAATTCCGATTAACGC-3′, 552 bp.

PCR conditions were as follows: initial denaturation at 94 °C for 5 min, 30 denaturation cycles at 94 °C for 40 s, annealing at 54 °C for 30 s, amplification at 72 °C for 1 min, and a final extension at 72 °C for 10 min. PCR amplification products were identified by 1% agarose gel electrophoresis, and positive products were sequenced by Sangon Bioengineering (Shanghai, China) Co., Ltd.

### 2.4. Drug Susceptibility Testing

The Kirby–Bauer disk diffusion method was used to test the susceptibility of the isolated strains to 16 kinds of antibacterial drugs. According to the Clinical and Laboratory Standards Institute (CLSI) standards, the drug susceptibility test results were judged in three forms: sensitivity (S), intermediate (I), and resistance (R). *Escherichia coli* ATCC 25922 was used as the quality control strain.

### 2.5. Detection of Resistance Genes

DNA was extracted from the 175 *E. coli* strains by the water boiling method. In total, 23 resistance genes of *E. coli* were detected by PCR as described previously. The primer sequences amplified fragment sizes and annealing temperatures are shown in Table S3, relating to aminoglycosides (*aadA*, *aph(3′)- II*, *aac2*, *aac4*), beta-lactams (*CTX-M*, *SHV*, *TEM*, *OXA*, *CMY-2*), tetracyclines (*Tet(A)*, *Tet(B)*, *Tet(M)*), fluoroquinolones (*qnrA*, *qnrB*, *qnrS*, *oqxA*, *oqxB*, *aac(6′)- Ib-cr*, *gyrA, gyrB*, *parC*), sulfonamides (*sul-1*, *sul-2*, *dfra*), polypeptides (*mcr-1*), and chloramphenicol (*flor*). The primers were synthesized, and PCR products were sequenced by Sangon Bioengineering (Shanghai, China) Co., Ltd.

### 2.6. Multilocus Sequence Typing

To investigate the prevalence of multidrug-resistant strains, 30 multidrug-resistant *E. coli* strains (SF: *n* = 20, NSF: *n* = 10) were selected from 175 *E. coli* strains for multilocus sequence typing. Primer sequences of the seven housekeeping genes of *E. coli* (*adk*, *fumC*, *icd*, *purA*, *gyrB*, *recA*, and *mdh*) were obtained from http://enterobase.warwick.ac.uk/species/ecoli/allele_st_search, accessed on 13 August 2023 [19,20]. The primer sequences, fragment sizes, and annealing temperatures are shown in Table S4. The sequencing results were spliced by SeqMan and uploaded to the database, generating an allelic map to determine the STs of *E. coli*. A phylogenetic tree was constructed and analyzed using Mega 7 software to analyze the genetic evolution.

### 2.7. Statistical Analysis

Statistical differences in AMR rates in *E. coli* isolates from two types of broiler farms were analyzed with the chi-square test and Fisher’s test by SPSS 25.0 (IBM Corporation, Somers, NY, USA). A value of probability (*p*) <0.05 was considered statistically significant.

## 3. Results

### 3.1. Isolation and Identification

**Figure 2:**
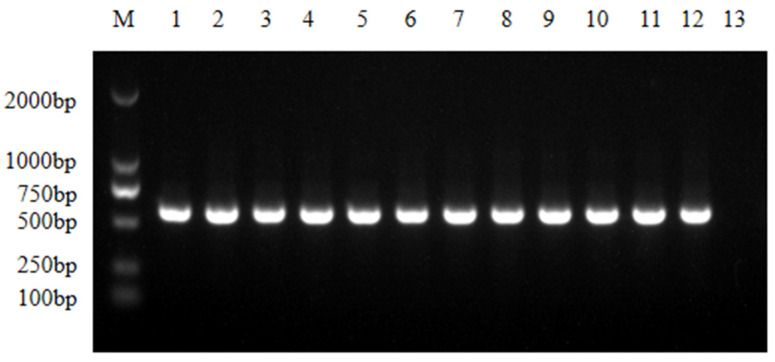
16S rRNA detection of partial *E. coli* isolate strains. M, DL2000DNA Marker; 1, ATCC25922; 2–12, partial *E. coli* isolate strains; 13, negative control.

A total of 175 strains (SF 115; NSF 60) were isolated and identified from 180 cloacal swab samples after separation and purification by MacConkey medium and eosin methylene blue medium, Gram staining morphological characteristics identification, and 16S rRNA PCR detection (Figure 2).

### 3.2. Results of Drug Resistance Phenotype Detection

The antibiotic sensitivity test results of 175 of the *E. coli* strains to 16 antibiotics are shown in Table 1. Both types of broiler farms had the highest rate of resistance to ampicillin, which was above 90.0%, and the lowest rate of resistance to meropenem, which was below 10.0%.

Based on the analysis, it was found that the *E. coli* strains in the SFs had the highest resistance rate to ampicillin (93.9%), followed by florfenicol, ceftiofur, enrofloxacin, sulfisoxazole, and cotrimoxazole, which were all over 70.0%. However, the resistance rates to ofloxacin, gentamicin, colistin, acetylmethaquine, ceftazidime, and meropenem were lower at 31.3%, 26.1%, 22.6%, 22.6%, 16.5%, and 7.0%, respectively. On the other hand, in the NSFs, the *E. coli* strains had a high resistance rate to florfenicol, ampicillin, and ceftiofur, with the resistance rate being over 90.0%. Additionally, the resistance rates for spectinomycin, apramycin, enrofloxacin, sulfisoxazole, and cotrimoxazole were above 70%. However, the resistance rates for colistin (28.3%), acetylmethaquine (13.3%), ceftazidime (16.7%), and meropenem (1.7%) were lower. These results indicated that the tested *E. coli* strains of the SFs and NSFs showed a high resistance rate to ampicillin and were sensitive to meropenem.

The analysis revealed that there were significant differences in the resistance rates of *E. coli* in the SFs and NSFs. Specifically, the resistance rates of gentamicin, spectinomycin, and ceftiofur were significantly lower in the SFs than in the NSFs (*p* < 0.05). Conversely, the resistance rate of cotrimoxazole was significantly lower in the NSFs than in the SFs (*p* < 0.05). This may be due to the extensive usage of cotrimoxazole before, resulting in relatively high drug resistance levels. Furthermore, the resistance to apramycin, tetracycline, florfenicol, ampicillin, ceftazidime, enrofloxacin, and colistin was lessened in the SFs than in the NSFs, although the difference was not statistically significant (*p* > 0.05). According to the study’s findings, the National Action Program for the Reduction of Veterinary Antimicrobial Drug Use has successfully reduced antibiotic resistance to a certain extent in Hebei Province, China. However, the resistance situation is still relatively serious, and there is still a need to continue long-term monitoring of antibiotic resistance in broiler farms.

### 3.3. Results of Multidrug Resistance of Isolated Strains

**Figure 3:**
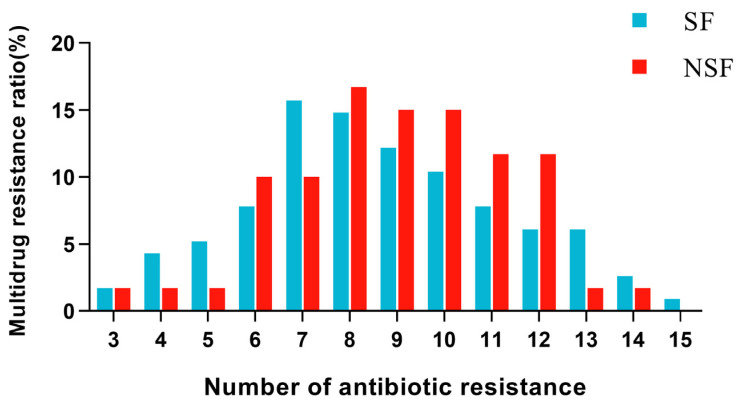
Multidrug resistance of isolated strains in the two types of broiler farms.

Most *E. coli* strains were MDR strains (96.57%, 169/175) resistant to three or more different types of antimicrobials. The MDR rates were different between the *E. coli* strains of the SFs (95.65%, 110/115) and NSFs (98.33%, 59/115). The 175 *E. coli* strains showed a broad multidrug-resistance pattern with 151 AMR patterns. The MDR strains of SFs (74.8%, 84/115) and NSFs (90.0%, 54/60) were both the majority resistant to 6–12 classes of antimicrobials (Figure 3). The *E. coli* strains of NSF have a more severe multidrug resistance situation, with the majority of *E. coli* strains being resistant to more types of antibiotics. Overall, the pilot work slightly alleviated the multidrug resistance of chicken-derived *E. coli* strains in Hebei Province, China.

### 3.4. Resistance Gene Detection Results

In this study, a total of 23 ARGs were detected (Table 2). All *E. coli* strains carried *aph(3′)-II* (100.0%), *gyrA* (100.0%), *gyrB* (100.0%), and *parC* (100.0%), while the detection rates of the *aac2*, *SHV*, *CMY-2*, *Tet (M)*, *qnrB*, *aac(6′)-Ib-cr*, and *dfra* genes were all below 20%. Moreover, in the SFs, the resistance genes *TEM* (97.4%), *Tet(A)* (88.7%), *oqxB* (84.3%), and *flor* (87.8%) were the prevalent genes, followed by *aac4* (70.4%), *qnrA* (75.7%), *OXA* (71.3%), *Tet(B)* (55.7%), *qnrs* (44.6%), *oqxA* (66.1%), *sul-1* (40.9%), and *mcr-1* (50.3%). In the NSFs, the resistance genes *aadA* (88.3%), *TEM* (96.7%), *Tet(A)* (88.3%), *qnrA* (91.7%), *sul-1* (83.3%), and *flor* (87.8%) were the prevalent genes, followed by the *aac4* (75.0%), *CTX-M* (63.3%), *OXA* (68.3%), *oqxA* (63.3%), *oqxB* (73.3%), and *sul-1* (48.3%) genes. The results of significant difference analysis showed that the detection rate of the *aac2* and *qnrA* genes of *E. coli* in the SFs was significantly lower than that of the NSFs (*p* < 0.05), while the *Tet(B)*, *mcr-1*, *CMY-2* and *qnrB* genes of *E. coli* in the SFs were significantly higher than those of the NSFs (*p* < 0.05).

The correlation between resistance genes and resistant phenotypes is shown in Table S4. In the present study, the *aadA, aph(3′)-II* and *aac4* genes primarily mediate aminoglycoside antibiotic resistance, which showed a greater than 80% consistency rate of drug resistance for these strains. *TEM* genes primarily mediate β-lactam antibiotic resistance, with an above 90% consistency rate of drug resistance. The consistency rate of *Tet(A)* genes with tetracycline resistance is above 90%. In terms of quinolone resistance, the *gyrA*, *gyrB*, and *parC* genes are the main mediators of quinolone antibiotic resistance, and the consistency rate is close to 100%. This means that almost all quinolone-resistant strains carry *gyrA*, *gyrB*, and *parC* resistance genes. Mequindox, colistin-E, and sulfonamide resistance is mainly due to the expression of the *oqxB*, *mcr-1,* and *sul-2* genes, respectively.

The resistance rates to 16 antibiotics were correlated with the detection rates of related resistance genes in two different types of broiler farms. Overall, there is a similar prevalence and diversity of 23 resistance genes in the *E. coli* strains of the two types of broiler farms in Hebei Province, China.

### 3.5. MLST Typing Results

Multilocus sequence typing (MLST) analysis showed that the 30 multidrug-resistant *E. coli* strains belonged to 18 ST types and one novel ST (Table 3).

**Figure 4:**
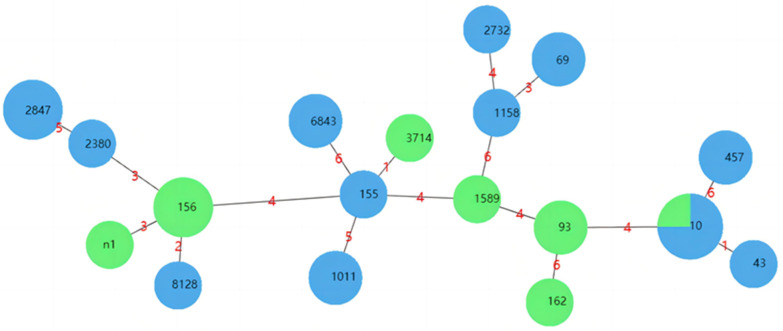
Cluster analysis was performed by geoBURST software v1.1.5. The black number represents ST types; The red number represents the difference in allele numbers between different ST types.

ST10 of *E. coli* strains was the dominant genotype (13.3%), and the percentages of ST156 and ST2847 were both 10.0%, followed by ST93, ST69, ST6843, ST457, and ST1011, which were all 6.67%, and the other 10 ST types were all 3.33%. There were differences in the distribution of *E. coli* ST types in the two types of broiler farms. In the SFs, the dominant STs were ST10 and ST2847, while in the NSFs, ST156 was the most common (Figure 4).

**Figure 5:**
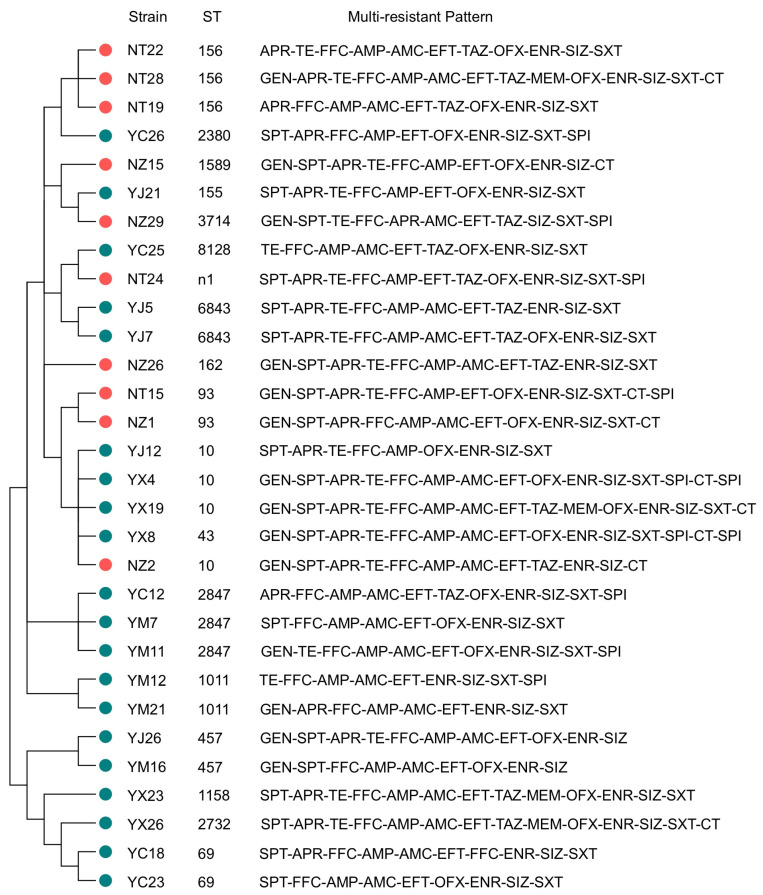
Phylogenetic tree of *E. coli* strains based on STs and multiresistance patterns. The red dots represent *E. coli* strains of SFs, and the blue dots represent *E. coli* strains of NSFs.

The cluster analysis results of 30 multidrug-resistant *E. coli* strains of two types of broiler farms using Mega 7.0 software showed (Figure 5) that there was a certain genetic relationship between the SFs and NSFs. Out of the 30 multidrug-resistant *E. coli* strains, there were 18 STs with 28 multidrug-resistant patterns. Notably, strains with the same ST may not share the same multidrug-resistance pattern; for instance, the ST10 strain YX4, ST43 strain YX8, ST2847 strain YM7, and ST69 strain YC23. There was no obvious correlation between the ST types of the strain and the MDR patterns.

## 4. Discussion

The increased use of antimicrobial medications in livestock and poultry production has resulted in a number of issues with antibiotic resistance [21]. A governmental initiative to lower the usage of veterinary antimicrobial medicines has been in place in China since 2018. In this study, we examined and analyzed the resistance to a total of 16 antimicrobial drugs in seven classes and the carriage of resistance genes from the two types of broiler farms (SF and NSF) in Hebei Province following the implementation of the National Veterinary Antimicrobial Drug Use Reduction Action Pilot Work Program. A differential analysis of the drug resistance in *E. coli* strains from the two types of broiler farms was performed.

Ampicillin resistance rates were found to be highest in *E. coli* strains in two types of broiler farms, exceeding 90.0%. Additionally, resistance rates to florfenicol, ceftiofur, enrofloxacin, and sulfisoxazole were over 70.0%. This is consistent with the results of domestic and foreign surveys in recent years. Similarly, high resistance to ampicillin, tetracycline, and sulfisoxazole was observed in chicken-derived *E. coli* strains of Shandong Province, China [22,23]. Likewise, most *E. coli* isolate strains displayed serious resistance to tetracycline, sulfamethoxazole, florfenicol, and ampicillin in Eastern China [24]. According to a report by Osman et al. [25], resistance to antibiotics such as ampicillin, tetracycline, erythromycin, sulfamethoxazole, and doxycycline and streptomycin is the most common. However, most of chicken-derived *E. coli* were strains showed highly sensitive to the ceftazidime and meropenem of β-lactams in this study. Similar results were observed in Zhejiang Province, China, where most isolates showed highly sensitive to ceftazidime (12.81%) and meropenem (0.75%), but serious resistance to tetracycline (92.92%), sulfisoxazole (93.05%), florfenicol (83.11%), and ampicillin (78.27%) [26], which is also similar to the previous reports in Jiangxi Province, China [27]. It has been observed that in France, Germany, Italy, and the United Kingdom, *E. coli* strains have a high resistance to ampicillin and cotrimoxazole, while resistance to ceftazidime and meropenem is very low [28]. Antibiotic resistance is closely linked to the use of antibiotics, and it has been found that food animals [29], especially chickens [30], are given high levels of antimicrobial drugs such as tetracyclines, fluoroquinolones, β-lactams, and aminoglycosides. Among β-lactams, amoxicillin, ampicillin, and ceftiofur are legally and extensively used, followed by fluoroquinolones such as norfloxacin and ofloxacin. This explains the high resistance levels to these antibiotics on broiler farms. Moreover, a study conducted between 2013 and 2019 revealed that the resistance and multiresistance of *E. coli* strains to important antibiotics for human medicine, such as third-generation cephalosporins and meropenem, have increased over time compared to 2013 [31]. This trend of antibiotic resistance is concerning. It is necessary to strictly control antibiotic use and monitor resistance to protect human and public health.

Bacterial resistance genotypes are determinants of the resistance phenotype. By identifying carriers of resistance genes in multidrug-resistant strains is crucial to support clinical rational antibiotic use and gain insight into antibiotic resistance gene epidemic characteristics [32]. Among the *E. coli* strains in this study, *aph(3′)-II*, *aadA*, *TEM*, *gyrA*, *gyrB*, *parC*, and *flor* resistance genes were highly prevalent with the detection rates all above 80%. The primary resistance mechanism of aminoglycosides is inactivated by aminoglycoside-modifying enzymes. Our study found that *E. coli* isolates were mainly resistant to aminoglycosides due to the presence of *aph(3′)-II* encoding aminoglycoside phosphotransferases (APHs) and *aadA* encoding aminoglycoside nucleotidyltransferase (ANTs). According to Liu et al., the highest detection rate for *aadA* (80%) was observed in chicken-derived *E. coli* strains in Shandong Province, China [33], while Song et al. noted that the detection frequencies of the *aphA3* resistance genes in chicken-derived *E. coli* strains were above 50% [34]. The *CTX-M* and *TEM* family of extended-spectrum beta-lactamases (ESBLs) has long been considered the most widespread globally, among the 262 ESBL-positive *E. coli* isolates in central China, *CTX-M* (97.33%) was the most prevalent type, followed by *TEM* (76.72%) [35,36]. Similarly, our study found that *CTX-M* and *TEM* primarily mediate β-lactam antibiotic resistance. In terms of quinolone resistance, *gyrA*, *gyrB*, and *parC* genes are the main contributors, followed by *qnrA*, *qnrB*, and *qnrS* genes. However, fluoroquinolone-modified acetyltransferase genes (*aac(6′)-Ib-cr*) are less common [37,38]. *Flor* genes encoding mainly mediate the florfenicol resistance, and it has been reported that among 106 florfenicol resistant *E. coli* strains, approximately 91.51% (97/106) of *E. coli* strains carry *flor* genes [39]. The key to antibiotic resistance is the effective expression of resistance genes [32,40]. We observed a correlation between the detection rates of related resistance genes and the resistance rates to 16 antibiotics in two different types of broiler farms. The resistance to a particular class of antibiotics increased with the detection rate of its related resistance genes. Specifically, the resistance rates of gentamicin, spectinomycin, and ceftiofur were significantly lower in the SFs than in the NSFs (*p* < 0.05). This may be because the NSF uses more β-lactams antibiotics in the early fattening stage and more aminoglycosides antibiotics in the terminal fattening stage than that in the SF, in addition, the overall level of antibiotic use in the SF is lower than that in the NSF. The results of significant difference analysis showed that the detection rates of the *aadA, aph(3′)-Ⅱ*, *aac2*, and *aac4* genes of *E. coli* in the SFs were lower than those of the NSFs, especially the detection rate of *aac2*, which significantly lower than that of the NSFs (*p* < 0.05). This may be related to the lower use of aminoglycoside antibiotics in the SF.

In this study, the antibiotic resistance in SFs decreased to a certain extent compared with NSFs in Hebei Province, China, but the difference in some antimicrobial drugs was not significant. On the one hand, this may be due to the short period of antimicrobial drug reduction in SFs (3–4 years), and the fact that antibiotics have been administered in poultry farming for more than 50 years, and the resistance genes remain at high levels for a long time, regardless of the antibiotic pressure; on the other hand, there are different medication habits in different regions [41]. Overall, serious antibiotic resistance problems still exist in the two types of broiler farms in Hebei Province, China, and the farms should further strengthen the rational use of antibiotics to effectively curb antibiotic resistance.

In recent years, multilocus sequence typing (MLST) technology has been widely used in bacterial typing, which can clarify the affinity and evolutionary relationship of strains and provide a reference for the epidemiological investigation, control, and prevention of *E. coli* [42,43]. It was found that 30 multidrug-resistant *E. coli* strains from two types of broiler farms were categorized into a total of 18 ST types, with ST10 as the dominant ST type, followed by ST156, ST2847, ST93, ST69, ST6843, ST457, and ST1011. In the SFs, the dominant STs were ST10 and ST2847, while in the NSFs, ST156 was the most common ST type. Zhou et al. [11] reported that among strains of porcine and avian origin, ST10 was the dominant ST type, followed by ST48, ST58, and ST162. A study of the MLST data showed that the *E. coli* strains of ST10, ST48, ST95, and ST117 were dominant in poultry [36,40,44], of which ST10 is predominant in human, poultry, and swine EXPEC at home and abroad, and it has been shown that the strains of ST10, ST95, ST23, ST117, and ST131 may cause zoonotic diseases [45]. Notably, strains with the same ST may not share the same multidrug-resistance pattern. Out of all the *E. coli* strains that were isolated, only the ST10 type showed a crossover between the SFs and NSFs. This suggests that the ST types have a complex and diverse genetic makeup. However, due to the limited number of samples and the short implementation period for antimicrobial reduction, it is unclear whether the implementation had a significant impact. Therefore, more monitoring and research are needed to fully understand the effects.

The limitation of this study is that only six farms were analyzed, which were further divided into two categories, with monitoring of antibiotic reduction (four farms) and without monitoring (two farms), and only 30 cloacal samples were analyzed per farm. In the future long-term studies, we will expand the sampling range and increase the number of samples, so as to make the conclusions of our study more abundant and reliable.

## 5. Conclusions

In this study, we investigated and analyzed the antibiotic resistance of *E. coli* strains and ST types of some multidrug-resistant *E. coli* strains from two types of broiler farms in Hebei Province. It was found that the implementation of antimicrobial reduction could reduce the resistance to certain antibiotics and curb the spread of antibiotic resistance. It is worthy studying the long-term effects of the implementation of antimicrobial reduction with a larger sample size in the future.

## Figures and Tables

**Table 1:** Resistance rates of isolates from two types of broiler farms to 16 antimicrobials.

Drug Type	Drug Name	Abbreviation	SF (%)	NSF (%)
Aminoglycosides	Gentamicin	GEN	26.1 ^A^	56.7 ^B^
	Spectinomycin	SPT	40.0 ^A^	71.7 ^B^
	Apramycin	APR	69.6	75.0
Tetracyclines	Tetracycline	TE	45.2	53.3
Chloramphenicol	Florfenicol	FFC	86.1	93.3
β-lactams	Ampicillin	AMP	93.9	98.3
	Amoxicillin/clavulanic acid	AMC	58.3	45.0
	Ceftiofur	EFT	82.6 ^a^	96.7 ^b^
	Ceftazidime	TAZ	16.5	16.7
	Meropenem	MEM	7.0	1.7
Quinolones	Ofloxacin	FOX	31.3	30.0
	Enrofloxacin	ENR	75.7	76.7
Sulfonamides	Sulfisoxazole	SIZ	79.1	71.7
	Cotrimoxazole	SXT	73.9 ^a^	55.0 ^b^
Peptides	Colistin-E	CT	22.6	28.3
Quinoxalines	Mequindox	SPI	22.6	13.3

Note: different uppercase letters on the shoulders of peer data indicate extremely significant differences (*p* < 0.01); different uppercase and lowercase letters on the shoulders indicate significant differences (*p* < 0.05); the same letters or no letters on the shoulders indicate no significant differences (*p* > 0.05).

**Table 2:** The detection rate of resistance genes of the two types of broiler farms.

Drug Type	Gene	SF (%)	NSF (%)
Aminoglycosides	*aadA*	78.3	88.3
	*aph(3′)-Ⅱ*	100.0	100.0
	*aac2*	13.0 ^A^	36.7 ^B^
	*aac4*	70.4	75.0
β-lactams	*CTX-M*	73.9	63.3
	*SHV*	16.5	8.3
	*TEM*	97.4	96.7
	*OXA*	71.3	68.3
	*CMY-2*	14.8 ^a^	1.7 ^b^
Tetracyclines	*Tet(A)*	88.7	88.3
	*Tet(B)*	55.7 ^A^	18.3 ^B^
	*Tet(M)*	10.4	8.3
Quinolones	*qnrA*	75.7 ^a^	91.7 ^b^
	*qnrB*	18.3 ^a^	8.3 ^b^
	*qnrs*	42.6	33.3
	*oqxA*	66.1	63.3
	*oqxB*	84.3	73.3
	*aac(6′)- Ib-cr*	10.4	11.7
	*gyrA*	100.0	100.0
	*gyrB*	100.0	100.0
	*parC*	100.0	100.0
Sulfonamides	*sul-1*	40.9	48.3
	*sul-2*	79.1	83.3
	*dfra*	7.0	3.3
Peptides	*mcr-1*	53.0 ^A^	28.3 ^B^
Chloramphenicol	*flor*	87.8	93.3

Note: different uppercase letters on the shoulders of peer data indicate extremely significant differences (*p* < 0.01); different uppercase and lowercase letters on the shoulders indicate significant differences (*p* < 0.05); the same letters or no letters on the shoulders indicate no significant differences (*p* > 0.05).

**Table 3:** Distribution of the ST type of the 30 isolates and multidrug resistance profile.

ST Type	Number	Strain Number	Farm Type	Multidrug Resistance Profile
ST10	4	YJ12	SF	SPT-APR-TE-FFC-AMP-OFX-ENR-SIZ-SXT
		NZ2	NSF	GEN-SPT-APR-TE-FFC-AMP-AMC-EFT-TAZ-ENR-SIZ-CT
		YX4	SF	GEN-SPT-APR-TE-FFC-AMP-AMC-EFT-OFX-ENR-SIZ-SXT-SPI-CT-SPI
		YX19	SF	GEN-SPT-APR-TE-FFC-AMP-AMC-EFT-TAZ-MEM-OFX-ENR-SIZ-SXT-CT
ST43	1	YX8	SF	GEN-SPT-APR-TE-FFC-AMP-AMC-EFT-OFX-ENR-SIZ-SXT-SPI-CT-SPI
ST155	1	YJ21	SF	SPT-APR-TE-FFC-AMP-EFT-OFX-ENR-SIZ-SXT
ST156	3	NT19	NSF	APR-FFC-AMP-AMC-EFT-TAZ-OFX-ENR-SIZ-SXT
		NT22	NSF	APR-TE-FFC-AMP-AMC-EFT-TAZ-OFX-ENR-SIZ-SXT
		NT28	NSF	GEN-APR-TE-FFC-AMP-AMC-EFT-TAZ-MEM-OFX-ENR-SIZ-SXT-CT
ST93	2	NZ1	NSF	GEN-SPT-APR-FFC-AMP-AMC-EFT-OFX-ENR-SIZ-SXT-CT
		NT15	NSF	GEN-SPT-APR-TE-FFC-AMP-EFT-OFX-ENR-SIZ-SXT-CT-SPI
ST162	1	NZ26	NSF	GEN-SPT-APR-TE-FFC-AMP-AMC-EFT-TAZ-ENR-SIZ-SXT
ST69	2	YC18	SF	SPT-APR-FFC-AMP-AMC-EFT-FFC-ENR-SIZ-SXT
		YC23	SF	SPT-FFC-AMP-AMC-EFT-OFX-ENR-SIZ-SXT
ST6843	2	YJ5	SF	SPT-APR-TE-FFC-AMP-AMC-EFT-TAZ-ENR-SIZ-SXT
		YJ7	SF	SPT-APR-TE-FFC-AMP-AMC-EFT-TAZ-OFX-ENR-SIZ-SXT
ST457	2	YJ26	SF	GEN-SPT-APR-TE-FFC-AMP-AMC-EFT-OFX-ENR-SIZ
		YM16	SF	GEN-SPT-FFC-AMP-AMC-EFT-OFX-ENR-SIZ
ST1589	1	NZ15	NSF	GEN-SPT-APR-TE-FFC-AMP-EFT-OFX-ENR-SIZ-CT
ST1158	1	YX23	SF	SPT-APR-TE-FFC-AMP-AMC-EFT-TAZ-MEM-OFX-ENR-SIZ-SXT
ST2732	1	YX26	SF	SPT-APR-TE-FFC-AMP-AMC-EFT-TAZ-MEM-OFX-ENR-SIZ-SXT-CT
ST2847	3	YM7	SF	SPT-FFC-AMP-AMC-EFT-OFX-ENR-SIZ-SXT
		YM11	SF	GEN-TE-FFC-AMP-AMC-EFT-OFX-ENR-SIZ-SXT-SPI
		YC12	SF	APR-FFC-AMP-AMC-EFT-TAZ-OFX-ENR-SIZ-SXT-SPI
ST1011	2	YM12	SF	TE-FFC-AMP-AMC-EFT-ENR-SIZ-SXT-SPI
		YM21	SF	GEN-APR-FFC-AMP-AMC-EFT-ENR-SIZ-SXT
ST8128	1	YC25	SF	TE-FFC-AMP-AMC-EFT-TAZ-OFX-ENR-SIZ-SXT
ST2380	1	YC26	SF	SPT-APR-FFC-AMP-EFT-OFX-ENR-SIZ-SXT-SPI
ST3714	1	NZ29	NSF	GEN-SPT-TE-FFC-APR-AMC-EFT-TAZ-SIZ-SXT-SPI
n1	1	NT24	NSF	SPT-APR-TE-FFC-AMP-EFT-TAZ-OFX-ENR-SIZ-SXT-SPI

## Data Availability

The study’s original contributions are included in the article; further inquiries can be directed to the corresponding authors.

## Supplementary Materials

The following supporting information can be downloaded at: https://www.mdpi.com/article/10.3390/ani13203194/s1, Table S1: Primer sequences of drug resistance genes; Table S2: Information on antibiotic treatments in the six sampling farms during the study period; Table S3: Primer sequences of drug resistance genes; Table S4: Primer sequences of housekeeping genes; Table S5: The consistency rate of resistant genes with resistant phenotypes.

## References

[1] Hopkins K.L., Davies R.H., Threlfall E.J. (2005). Mechanisms of Quinolone Resistance in *Escherichia coli* and Salmonella: Recent Developments. Int. J. Antimicrob. Agents.

[2] Castrignanò E., Kannan A.M., Proctor K., Petrie B., Hodgen S., Feil E.J., Lewis S.E., Lopardo L., Camacho-Muñoz D., Rice J. (2020). (Fluoro)quinolones and quinolone resistance genes in the aquatic environment: A river catchment perspective. Water Res..

[3] Yang Y., Zhou R., Chen B., Zhang T., Hu L., Zou S. (2018). Characterization of Airborne Antibiotic Resistance Genes from Typical Bioaerosol Emission Sources in the Urban Environment Using Metagenomic Approach. Chemosphere.

[4] Costa D., Poeta P., Sáenz Y., Coelho A.C., Matos M., Vinué L., Rodrigues J., Torres C. (2008). Prevalence of Antimicrobial Resistance and Resistance Genes in Faecal *Escherichia coli* Isolates Recovered from Healthy Pets. Vet. Microbiol..

[5] Plata G., Baxter N.T., Susanti D., Volland-Munson A., Gangaiah D., Nagireddy A., Mane S.P., Balakuntla J., Hawkins T.B., Kumar Mahajan A. (2022). Growth Promotion and Antibiotic Induced Metabolic Shifts in the Chicken Gut Microbiome. Commun. Biol..

[6] Paul S.S., Rama Rao S.V., Hegde N., Williams N.J., Chatterjee R.N., Raju M., Reddy G.N., Kumar V., Phani Kumar P.S., Mallick S. (2022). Effects of Dietary Antimicrobial Growth Promoters on Performance Parameters and Abundance and Diversity of Broiler Chicken Gut Microbiome and Selection of Antibiotic Resistance Genes. Front. Microbiol..

[7] Chokshi A., Sifri Z., Cennimo D., Horng H. (2019). Global Contributors to Antibiotic Resistance. J. Glob. Infect. Dis..

[8] Qiao M., Ying G.G., Singer A.C., Zhu Y.G. (2018). Review of Antibiotic Resistance in China and Its Environment. Environ. Int..

[9] Tang B., Wang J., Zheng X., Chang J., Ma J., Wang J., Ji X., Yang H., Ding B. (2022). Antimicrobial Resistance Surveillance of *Escherichia coli* from Chickens in the Qinghai Plateau of China. Front. Microbiol..

[10] Pulingam T., Parumasivam T., Gazzali A.M., Sulaiman A.M., Chee J.Y., Lakshmanan M., Chin C.F., Sudesh K. (2022). Antimicrobial Resistance: Prevalence, Economic Burden, Mechanisms of Resistance and Strategies to Overcome. Eur. J. Pharm. Sci..

[11] Zhou W., Lin R., Zhou Z., Ma J., Lin H., Zheng X., Wang J., Wu J., Dong Y., Jiang H. (2022). Antimicrobial Resistance and Genomic Characterization of *Escherichia coli* from Pigs and Chickens in Zhejiang, China. Front. Microbiol..

[12] Zhang Q.Q., Ying G.G., Pan C.G., Liu Y.S., Zhao J.L. (2015). Comprehensive Evaluation of Antibiotics Emission and Fate in the River Basins of China: Source Analysis, Multimedia Modeling, and Linkage to Bacterial Resistance. Environ. Sci. Technol..

[13] Manyi-Loh C., Mamphweli S., Meyer E., Okoh A. (2018). Antibiotic Use in Agriculture and Its Consequential Resistance in Environmental Sources: Potential Public Health Implications. Molecules.

[14] Sabino Y.N.V., Santana M.F., Oyama L.B., Santos F.G., Moreira A.J.S., Huws S.A., Mantovani H.C. (2019). Characterization of Antibiotic Resistance Genes in the Species of the Rumen Microbiota. Nat. Commun..

[15] Opatowski L., Opatowski M., Vong S., Temime L. (2021). A One-Health Quantitative Model to Assess the Risk of Antibiotic Resistance Acquisition in Asian Populations: Impact of Exposure Through Food, Water, Livestock and Humans. Risk Anal..

[16] Tiseo K., Huber L., Gilbert M., Robinson T.P., Van Boeckel T.P. (2020). Global Trends in Antimicrobial Use in Food Animals from 2017 to 2030. Antibiotics.

[17] Mendelson M., Matsoso M.P. (2015). The World Health Organization Global Action Plan for Antimicrobial Resistance. S. Afr. Med. J..

[18] Ying G.G., He L.Y., Ying A.J., Zhang Q.Q., Liu Y.S., Zhao J.L. (2017). China Must Reduce Its Antibiotic Use. Environ. Sci. Technol..

[19] Zhou Z., Alikhan N.F., Mohamed K., Fan Y., Achtman M. (2020). The Enterobase User’s Guide, with Case Studies on Salmonella Transmissions, Yersinia Pestis Phylogeny, and Escherichia Core Genomic Diversity. Genome Res..

[20] Wirth T., Falush D., Lan R., Colles F., Mensa P., Wieler L.H., Karch H., Reeves P.R., Maiden M.C., Ochman H. (2006). Sex and Virulence in *Escherichia coli*: An Evolutionary Perspective. Mol. Microbiol..

[21] O’Brien T.F. (2002). Emergence, Spread, and Environmental Effect of Antimicrobial Resistance: How Use of an Antimicrobial Anywhere Can Increase Resistance to Any Antimicrobial Anywhere Else. Clin. Infect. Dis..

[22] Han T., Zhang Q., Liu N., Wang J., Li Y., Huang X., Liu J., Wang J., Qu Z., Qi K. (2020). Changes in Antibiotic Resistance of *Escherichia coli* During the Broiler Feeding Cycle. Poult. Sci..

[23] Zhao X., Liu Z., Zhang Y., Yuan X., Hu M., Liu Y. (2020). Prevalence and Molecular Characteristics of Avian-Origin Mcr-1-Harboring *Escherichia coli* in Shandong Province, China. Front. Microbiol..

[24] Afayibo D.J.A., Zhu H., Zhang B., Yao L., Abdelgawad H.A., Tian M., Qi J., Liu Y., Wang S. (2022). Isolation, Molecular Characterization, and Antibiotic Resistance of Avian Pathogenic *Escherichia coli* in Eastern China. Vet. Sci..

[25] Osman K.M., Kappell A.D., Elhadidy M., ElMougy F., El-Ghany W.A.A., Orabi A., Mubarak A.S., Dawoud T.M., Hemeg H.A., Moussa I.M.I. (2018). Poultry Hatcheries as Potential Reservoirs for Antimicrobial-Resistant *Escherichia coli*: A Risk to Public Health and Food Safety. Sci. Rep..

[26] Ma J., Zhou W., Wu J., Liu X., Lin J., Ji X., Lin H., Wang J., Jiang H., Zhou Q. (2022). Large-Scale Studies on Antimicrobial Resistance and Molecular Characterization of *Escherichia coli* from Food Animals in Developed Areas of Eastern China. Microbiol. Spectr..

[27] Tan M.F., Li H.Q., Yang Q., Zhang F.F., Tan J., Zeng Y.B., Wei Q.P., Huang J.N., Wu C.C., Li N. (2023). Prevalence and Antimicrobial Resistance Profile of Bacterial Pathogens Isolated from Poultry in Jiangxi Province, China from 2020 to 2022. Poult. Sci..

[28] De Jong A., El Garch F., Hocquet D., Prenger-Berninghoff E., Dewulf J., Migura-Garcia L., Perrin-Guyomard A., Veldman K.T., Janosi S., Skarzynska M. (2022). European-Wide Antimicrobial Resistance Monitoring in Commensal *Escherichia coli* Isolated from Healthy Food Animals between 2004 and 2018. J. Antimicrob. Chemother..

[29] Silva A., Silva V., Pereira J.E., Maltez L., Igrejas G., Valentão P., Falco V., Poeta P. (2023). Antimicrobial Resistance and Clonal Lineages of *Escherichia coli* from Food-Producing Animals. Antibiotics.

[30] Liao M., Wu J., Li Y., Lu X., Tan H., Chen S., Huang W., Lian X., Sun J., Liao X. (2022). Prevalence and Persistence of Ceftiofur-Resistant *Escherichia coli* in a Chicken Layer Breeding Program. Animals.

[31] Sodagari H.R., Varga C. (2023). Evaluating Antimicrobial Resistance Trends in Commensal *Escherichia coli* Isolated from Cecal Samples of Swine at Slaughter in the United States, 2013–2019. Microorganisms.

[32] Rahman M.M., Husna A., Elshabrawy H.A., Alam J., Runa N.Y., Badruzzaman A.T.M., Banu N.A., Al Mamun M., Paul B., Das S. (2020). Isolation and Molecular Characterization of Multidrug-Resistant *Escherichia coli* from Chicken Meat. Sci. Rep..

[33] Liu C., Wang P., Dai Y., Liu Y., Song Y., Yu L., Feng C., Liu M., Xie Z., Shang Y. (2021). Longitudinal Monitoring of Multidrug Resistance in *Escherichia coli* on Broiler Chicken Fattening Farms in Shandong, China. Poult. Sci..

[34] Song Y., Yu L., Zhang Y., Dai Y., Wang P., Feng C., Liu M., Sun S., Xie Z., Wang F. (2020). Prevalence and Characteristics of Multidrug-Resistant Mcr-1-Positive *Escherichia coli* Isolates from Broiler Chickens in Tai’an, China. Poult. Sci..

[35] Wang Z., Lu Q., Mao X., Li L., Dou J., He Q., Shao H., Luo Q. (2022). Prevalence of Extended-Spectrum β-Lactamase-Resistant Genes in *Escherichia coli* Isolates from Central China during 2016–2019. Animals.

[36] Liu Z., Wang K., Zhang Y., Xia L., Zhao L., Guo C., Liu X., Qin L., Hao Z. (2021). High Prevalence and Diversity Characteristics of Bla(Ndm), Mcr, and Bla(Esbls) Harboring Multidrug-Resistant *Escherichia coli* from Chicken, Pig, and Cattle in China. Front. Cell. Infect. Microbiol..

[37] Nhung N.T., Chansiripornchai N., Carrique-Mas J.J. (2017). Antimicrobial Resistance in Bacterial Poultry Pathogens: A Review. Front. Vet. Sci..

[38] Awad A., Arafat N., Elhadidy M. (2016). Genetic Elements Associated with Antimicrobial Resistance among Avian Pathogenic *Escherichia coli*. Ann. Clin. Microbiol. Antimicrob..

[39] Li P., Zhu T., Zhou D., Lu W., Liu H., Sun Z., Ying J., Lu J., Lin X., Li K. (2020). Analysis of Resistance to Florfenicol and the Related Mechanism of Dissemination in Different Animal-Derived Bacteria. Front. Cell. Infect. Microbiol..

[40] Aworh M.K., Kwaga J.K.P., Hendriksen R.S., Okolocha E.C., Thakur S. (2021). Genetic Relatedness of Multidrug Resistant *Escherichia coli* Isolated from Humans, Chickens and Poultry Environments. Antimicrob. Resist. Infect. Control.

[41] Benameur Q., Gervasi T., Dahloum L., Rechidi-Sidhoum N., Boutaiba Benklaouz M., Yakubu A. (2023). Multidrug-Resistant *Escherichia coli* Isolated from Cleaned and Disinfected Poultry Houses Prior to Day-Old Chick Placement. J. Environ. Qual..

[42] Feil E.J., Enright M.C. (2004). Analyses of Clonality and the Evolution of Bacterial Pathogens. Curr. Opin. Microbiol..

[43] Wang Y., Zhou J., Li X., Ma L., Cao X., Hu W., Zhao L., Jing W., Lan X., Li Y. (2020). Genetic Diversity, Antimicrobial Resistance and Extended-Spectrum Β-Lactamase Type of *Escherichia coli* Isolates from Chicken, Dog, Pig and Yak in Gansu and Qinghai Provinces, China. J. Glob. Antimicrob. Resist..

[44] Lu Q., Zhang W., Luo L., Wang H., Shao H., Zhang T., Luo Q. (2022). Genetic Diversity and Multidrug Resistance of Phylogenic Groups B2 and D in Inpec and Expec Isolated from Chickens in Central China. BMC Microbiol..

[45] Danzeisen J.L., Wannemuehler Y., Nolan L.K., Johnson T.J. (2013). Comparison of Multilocus Sequence Analysis and Virulence Genotyping of *Escherichia coli* from Live Birds, Retail Poultry Meat, and Human Extraintestinal Infection. Avian Dis..

