# Supplementary material for: Identification, Typing, and Drug Resistance Analysis of Escherichia coli in Two Different Types of Broiler Farms in Hebei Province
Source: Animals (Basel). 2023 Oct 13;13(20):3194. doi: 10.3390/ani13203194 (PMC10603750; doi:10.3390/ani13203194)
Supplement: Supplementary file 1 [file animals-13-03194-s001.zip › Supplementary Materials.pdf]

Table S1. Characteristics of the six broiler chicken farms in Hebei Province, China.

	Farm 1	Farm 2	Farm 3	Farm 4	Farm 5	Farm 6
Type	SF	SF	SF	SF	NSF	NSF
geographical location	Chengde	Cangzhou	Tangshan	Baoding	Tangshan	Baoding
Latitude and longitude	117.517391°E, 41.059622°N	117.348791°E ,38.157693°N	117.934846°E ,40.185948°N	114.684007°E ,39.431477°N	118.664675°E ,39.52271°N	115.566786°E ,39.412227°N
Number of barn	6	8	12	9	10	12
Fattening Scale per barn	1500 m^2	1440 m^2	1600 m^2	1600 m^2	1600 m^2	1600 m^2
Animal quantity per barn (Unit: thousand)	40	30	30	30	33	30
Chicken breed	Arbor Acres broiler	Arbor Acres broiler	Arbor Acres broiler	Arbor Acres broiler	Arbor Acres broiler	Arbor Acres broiler
Animal habitat	Cages	Cages	Cages	Cages	Cages	Cages
Ventilation	Draught fans	Draught fans	Draught fans	Draught fans	Draught fans	Draught fans
Feeding patterns	Automatic	Automatic	Automatic	Automatic	Automatic	Automatic

Note: Two different types of farms were distinguished according to the requirements of the National Action Plan for the Reduction of the Use of Veterinary Antimicrobial Agents (2021-2025) (Document No. 31 [2021]) of the Ministry of Agriculture and Rural Affairs of China. The effectiveness of reducing antibiotic use in broiler farms is evaluated based on four aspects: the farm's basic conditions, regulations, relevant records, and the efficacy of reduction actions. The farm is inspected, scored, and evaluated, If the score is 80 or higher, it is considered a "Standard Farm" (SF), while scores below 80 are considered a "Non-Standard Farm" (NSF). The basic requirements for the use of antimicrobial agents in SFs are as follows: the amount of antimicrobial agents used per ton of broiler (gross weight) should be limited to 100g over a growth period of no more than 60 days.

Table S2. Information on antibiotic treatments in the 6 sampling farms during the study period.

	SF 1	SF 2	SF 3	SF 4	NSF 1	NSF 2
1-5	Lincomycin	Florfenicol	Cefalexin	Ampicillin	Ceftiofur;	Cefalexin
7-10	-	-	-	-	Enrofloxacin	-
12-15	-	-	-	-	-	Ceftriaxone; Levofloxacin
18-21	Sulfachloropyrazine sodium; Neomycine	Apramycine	-	Florfenicol	Timicosin; Doxycycline	-
26-29	-	-	Levofloxacin; Ceftriaxone	-	-	-
32-35	Lincomycin，Doxycycline	-	-	-	Kanamycin, Erythromycin	Lincomycin，Amikacin

Table S3. Primer sequences of drug resistance genes.

Drug type	Gene	Primer sequence (5'→3')	Size (bp)	Annealing temperature (℃)
Aminoglycosides	*aad A*	F: GCAGCGCAATGACATTCTTG<br>R: ATCCTTCGGCGCGATTTTG	282	60
	aph(3') - *II*	F: TGACTGGGCACAACAGACAA<br>R: CGGCGATACCGTAAAGCAC	677	55
	aac2	F: ACCCTACGAGGAGACTCTGAATG<br>R: CCAAGCATCGGCATCTCATA	384	56
	aac4	F: CTTCAGGATGGGCAAGTTGGT<br>R: TCATCTCGTTCTCCGCTCAT	286	60
β-lactams	*CTX-M*	F: AGTGAAAGCGAACCGAATC<br>R: CTGTCACAATGCTTTACC	365	55
	SHV	F: ATGCGTATATTCGCCTGTG<br>R: CCTCATTCAGTTCCGTTTCC	502	55
	TEM	F: ATTCTTGAAGACGAAAGGGC<br>R: ACGCTCAGTGGAACGAAAAC	1150	60
	OXA	F: ACACAATACATATCAACTTCGC<br>R: AGTGTGTTTAGAATGGTGATC	813	61
	CMY-2	F: ACAGCCTCTTTCTCCACA<br>R: ATTGCCTCTTCGTAACTCA	545	53
Tetracyclines	*Tet(A)*	F: GGCCTCAATTTCCTGACG<br>R: AAGCAGGATGTAGCCTGTGC	372	55
	Tet(B)	F: CATTAATAGGCGCATCGCTG<br>R: TGAGGTCATCGATAGCAGG	392	53
	Tet(M)	F: ACAGAAAGCTTATTTATAAC<br>R: TGGCGTGTCTATGATGTTCAC	171	55
Quinolones	*qnrA*	F: CAGCAAGAGGATTTCTCA<br>R: GGCAGCACTATTACTCCCAA	500	55
	qnrB	F; GATCGTGAAAGCCAGAAAG<br>R: ACGATGCCTGGTAGTTGTCC	395	53
	qnrs	F: GACGTGCTAACTTGCGTGAT<br>R: AACACCTCGACTTAAGTCTGA	388	57
	oqxA	F: CTCGGCGCGATGATGCT<br>R: CCACTCTTCACGGGAGACGA	392	55
	oqxB	F: CTCCCCCGGCGGGAAGTAC<br>R: CTCGGCCATTTTGGCGCGTA	512	53
	aac(6')-Ib-cr	F: TATGAGTGGCTAAATCGA<br>R: CCCGCTTTCTCGTAGCA	394	52
	gyrA	F: CTGCGCGGCTGTGTTATAATT<br>R: CCGTGCCGTCATAGTTATCAA	521	53
	gyrB	F: CTGCCGGGAAACTGGCAGA<br>R: TCGACGTCCGCATCGGTCAT	299	55
	parC	F: GTATGCGATGTCTGAACT<br>R: TTCGGTGTAACGCATTGC	230	56
Sulfonamides	*sul-1*	F: GTGACGGTGTTCGGCATTCT<br>R: TCCGAGAAGGTGATTGCGCT	779	58
	sul-2	F: CGGCATCGTCAACATAACCT<br>R: TGTGCGGATGAAGTCAGCTC	721	55

	dfra	F: GGAGTGCCAAAGGTGAACAGC<br>R: GAGGCGAAGTCTTGGGTAAAAAC	367	48
Peptides	*mcr-1*	F: CGGTCAGTCCGTTTGTTC<br>R: CTTGGTCGGTCTGTAGGG	309	55
Chloramphenicol	*flor*	F: GTCGAGAAATCCCATGAGTTCA<br>R: CAGACAGGATACCGACATTCAC	1645	58

Table S4. Primer sequences of housekeeping genes.

**Gene**	**Primer sequence (5'→3')**	**Size (bp)**	**Annealing temperature (°C)**
adk	F: ATTCTGCTTGGCGCTCCGGG<br>R: CCGTCAACTTTCGCGTATTT	583	52
fumC	F: TCACAGGTCGCCAGCGCTTC<br>R: GTACGCAGCGAAAAAAGATTC	806	52
icd	F: ATGGAAAGTAAAGTAGTTGTTCCGGCACA<br>R: GGACGCAGCAGGATCTGTT	878	52
purA	F: CGCGCTGATGAAAGAGATGA<br>R: CATACGGTAAGCCACGCAGA	816	54
gyrB	F: TCGGCGACACGGATGACGGC<br>R: ATCAGGCCTTCACGCGCATC	911	58
recA	F: CGCATTCGCTTTACCCTGACC<br>R: TCGTCGAAATCTACGGACCGGA	780	58
mdh	F: ATGAAAGTCGCAGTCCTCGGCGCT GCTGGCGG<br>R: TTAACGAACTCCTGCCCCAGAGCGA TATCTTTCTT	932	58

Table S5. The consistency rate of resistant genes with resistant phenotypes.

	Gentamicin	Spectinomycin	Apramycin	Ampicillin	Amoxicillin/ clavulanic	Ceftiofur	Ceftazidime	Meropenem
aadA	0.8/0.97	0.98/1	0.85/0.96					
aph3	1/1	1/1	1/1					
acc2	0.27/0.26	0.042/0.30	0.11/0.31					
acc4	0.90/0.91	0.93/0.81	1/0.96					
CTX-M				0.71/0.64	0.78/0.59	0.8/0.66	0.84/0.2	0.75/0
SHV				0.13/0.08	0.13/0	0.13/0.09	0/0.1	0/0
TEM				0.98/0.97	0.97/1	0.98/0.97	0.95/1	0.88/1
OXA				0.75/0.69	0.81/0.59	0.74/0.69	0.84/0.6	1/0
CMY-2				0.10/017	0.10/0.04	0.05/0.02	0.21/0	0/0

	Tetracycline	Florfenicol	Ofloxacin	Enrofloxacin	Sulfisoxazole	Cotrimoxazole	Colistin-E	Mequindox
Tet(A)	0.92/1							
Tet(B)	0.5/0.094							
Tet(M)	0.15/0.03							
flor		0.97/1						
qnrA			0.61/1	0.75/1				
qnrB			0.22/0.17	0.14/0.11				
qnrs			0.61/0.33	0.38/0.17				
oqxA			0.69/0.61	0.72/0.54				1/1
oqxB			0.86/0.67	0.82/0.67				0.73/0.88
acc(6')-cr-ib			0.06/0	0.11/0.15				
gyrA			1/1	0.98/1				
gyrB			1/1	0.98/1				
parC			1/1	0.98/1				
sul-1					0.46/0.58	0.47/0.52		
sul-2					0.91/0.89	0.89/0.82		
dfra					0.08/0.02	0.09/0.06		
mcr-1							0.77/0.533	

Note: The red represents SF, and the black represents NSF.
